# Rule-based generalization and peak shift in the presence of simple relational rules

**DOI:** 10.1371/journal.pone.0203805

**Published:** 2018-09-14

**Authors:** Jessica C. Lee, Evan J. Livesey

**Affiliations:** School of Psychology, University of Sydney, Sydney, NSW, Australia; Florida International University, UNITED STATES

## Abstract

After discrimination learning between two stimuli that lie on a continuum, animals typically exhibit generalization on the basis of similarity to the physical features of the stimuli, often producing a peak-shifted gradient. However, post-discrimination generalization in humans usually resembles a monotonically increasing (e.g., linear) gradient that is better characterized as following a relational rule describing the difference between the stimuli. The current study tested whether rule-based generalization could be disrupted by reducing the applicability of a relational rule on test. We compared generalization following a difficult categorization task between a group who could use their rule consistently throughout test (Group Consistent), and a group who could only apply their rule effectively on 50% of test trials and thus could only use it inconsistently (Group Inconsistent). Across two experiments, a peak shift was found in the Inconsistent group and a monotonic gradient in the Consistent group. A post-hoc sequential analysis revealed that the Inconsistent group produced both peak-shifted and monotonic gradients as a function of whether or not the relevant rule was applicable on the previous trial. Reducing the applicability of a rule on test thus appeared to lead participants to revert to generalizing on the basis of similarity. Our results suggest that humans learn about the physical features of the stimuli alongside relational rules, and that rule- and similarity-based learning can interact in determining generalization.

## Introduction

Generalization is a fundamental ability that allows humans and animals to adapt effectively to novel situations. A wide range of studies using simple discrimination tasks suggest that humans are capable of generalizing on the basis of similarity to the physical features of the stimuli, but also on the basis of the abstract relationships between stimuli (e.g., [1,2]). In contrast, there is still debate about the presence of even simple forms of relational generalization in infrahuman animals ([2]). Relational and feature-based generalization appear to encapsulate two different forms of learning in terms of their core content, but whether they represent the operation of independent learning processes is more difficult to determine empirically. Characterizing the nature of *interaction* between relational and feature-based generalization may help to address this issue.

One procedure where clear differences can emerge between relational and feature- or similarity-based responding is dimensional generalization and in particular, the phenomenon of peak shift ([3]). As we will discuss, peak shift is particularly suited to investigating a potential interaction between associative and rule-based processes since it is readily predicted by associative theories, easily contrasted with rule-based generalization, and emerges under conditions where rule use is hindered. In animal learning experiments, generalization is often examined by rewarding subjects for responding to a stimulus (S+), for example, a light of a particular wavelength or a tone of a particular frequency, and then assessing responding to stimuli with varying values along that same dimension of interest (e.g., [4,5]). Such studies in animals typically produce a peaked generalization gradient, with the highest rates of responding at the trained S+, and responding decreasing as a function of the degree of similarity between the test stimulus and the S+ (see [6], for a review). These peaked generalization gradients can be explained by assuming that behavior towards a stimulus is governed by the degree of similarity or shared features between the novel versus trained instances. This assumption is a fundamental tenet of many associative learning theories ([7,8,9,10,11,12,13]).

If instead of training responses to a single stimulus, animals are trained using differential reinforcement to discriminate between an S+ and S- that lie close together on the relevant dimension, a phenomenon called peak shift often occurs ([3]). Peak shift describes the situation in which the peak of the generalization gradient shifts from the location of the S+ in the direction away from the S-. This effect is well accounted for by associative models that employ elemental representation and conceive of the S+ and S- as a series of overlapping elements on a continuum ([7,10,14], but see [15], for an alternative rule-based explanation). Peak shift can be predicted if it is assumed that the S+ and S- activate elements on the continuum in a graded (Gaussian) manner during discrimination training. If the S+ and S- contain many common elements, a large proportion of elements on the dimension will be activated by both stimuli and accrue weak associative strength due to excitatory learning on S+ countermanded by inhibitory learning on S- trials. Units with the greatest difference in activation between the S+ and S- will thus accrue the strongest associative strength. Therefore, elemental theories directly predict that a stimulus slightly removed from the S+ in the direction away from the S- should accrue the most associative strength and produce the peak shift phenomenon.

A similar analysis (e.g., see [16]) can be applied for a discrimination task with two stimuli (i.e. S1 and S2) with competing corresponding responses (i.e. R1 and R2). These responses have a mutually exclusive relationship (if one is correct, the other is not), meaning that S1 serves as a S- to S2, and vice versa. Assuming that the probability of choosing the associated response is a function of the activation of that response relative to activation of the alternative response ([17]), the highest rates of R1 will be to a stimulus slightly displaced from S1 in the direction away from S2, and vice versa for S2, producing a two-way peak shift ([16]). In both differential and two-choice discrimination tasks, even when a shift in the peak of responding occurs, a decline in responding is usually observed for extreme stimuli that are sufficiently different from those used in training (e.g., [3], see [6]). This suggests that generalization still occurs on the basis of physical similarity, albeit with a learned bias towards exaggerated versions of the S+. Thus, a generalization gradient with a localized peak of responding is typically interpreted as evidence of generalization based on the number of shared features between the test and training stimulus (i.e. physical similarity), consistent with an associative analysis.

**Fig 1:**
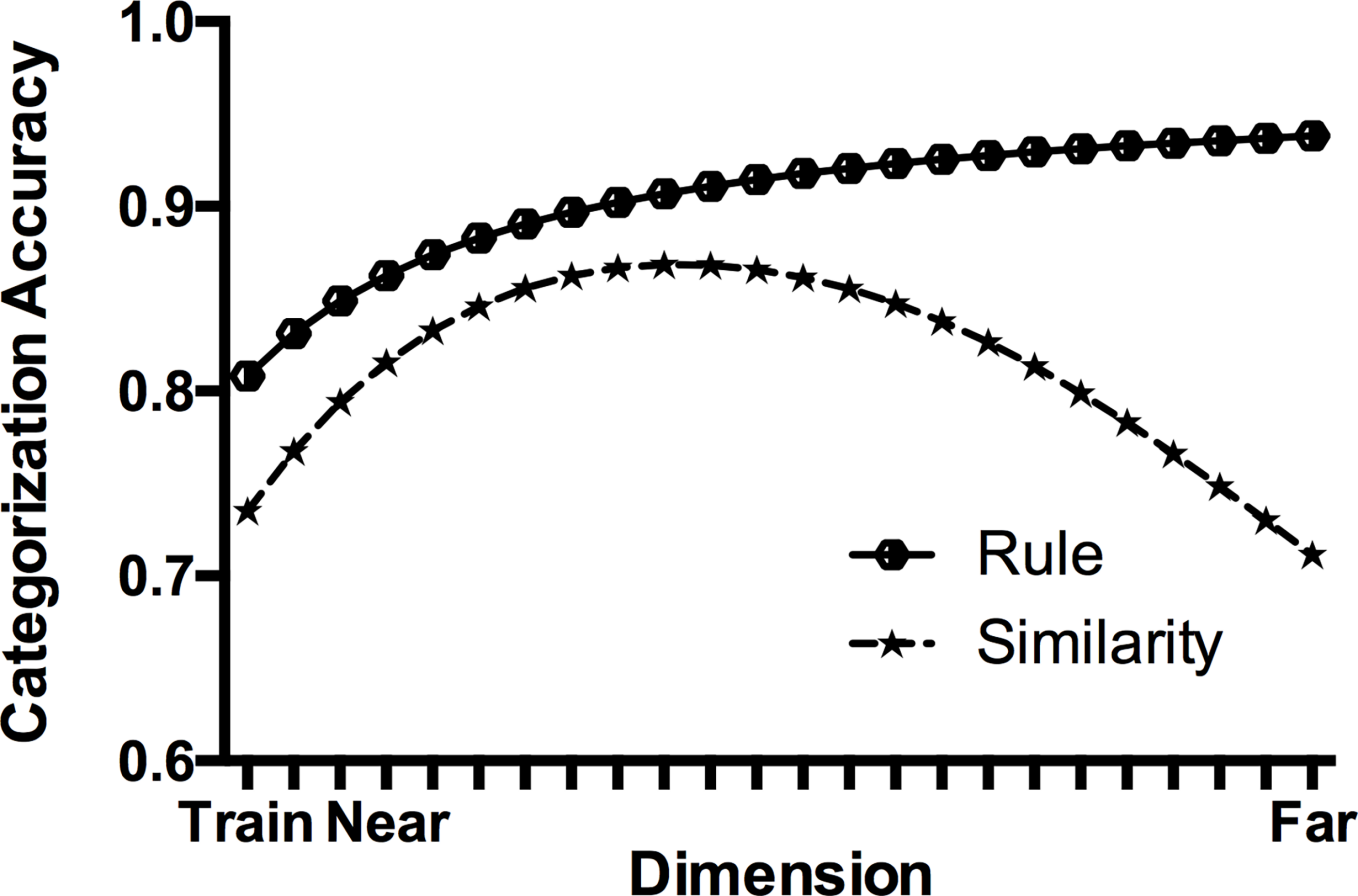
Example of rule-based and similarity-based generalization gradients in a two-choice categorization task. Note that the dimension has been folded such that the ‘Far’ point represents both extremes of the stimulus dimension (e.g., the brightest and darkest stimuli).

While peak shift is readily found in animal studies given appropriate parameters (see [18]), its elusiveness in human studies suggests there may be other important factors that dictate the manner of generalization. Analogous procedures in humans, particularly those involving simple forms of category or discrimination learning, often result in a different pattern of generalization. Instead of a peaked gradient, humans show a monotonically increasing gradient with the highest levels of responding or accuracy at the extreme ends of the dimension, where the stimuli are the most dissimilar from the training stimuli (e.g., [19,20,21,22], see Fig 1). If participants have formed a simple relational rule (e.g., concerning brightness: “category 1 is darker than category 2”), accuracy should be greatest at the extreme ends of the dimension (e.g., the lightest and darkest stimuli) since a relational rule is presumably easiest to apply at these test stimuli (in Fig 1, note that the dimension is folded such that the stimuli on the right represent the lightest *and* darkest stimuli). A monotonic gradient thus implies that participants have learnt about the relationships between the features of the stimuli rather than just the physical features themselves. Forming a relational rule requires abstract representations of the *difference* between the stimuli and thus the manner in which they are related to one another. Higher-order cognitive processes such as hypothesis-testing and reasoning are thought to be critical in the successful discovery of such rules ([2,23]). Therefore, the underlying mechanisms in rule-based generalization may be qualitatively different to the processes that support feature-based generalization in other animals (e.g., [24]). Even if rule- and feature-learning arise from the same system (e.g., [25]), it is clear that the content of learning differs, and that they produce distinct patterns of generalization.

There are, however, a few instances of peak shift reported in the human generalization literature. The conditions under which these studies have obtained peak shift are suggestive of a common feature-driven mechanism of generalization between humans and animals ([26]). For example, peak shift can be found when deriving a relational rule is difficult due to the complexity of the stimuli ([22]). Other demonstrations of peak shift in discrimination learning rely on using speeded responses during training with the stimuli presented as incidental cues ([27]), degrading the contingency between the training stimuli and the correct response ([28]), or interleaving two qualitatively distinct sets of stimuli to decrease the opportunity for stimulus comparison between trials ([20]). Taken together, these demonstrations suggest that when the training stimuli and procedures minimize the opportunity to form a relational rule, humans generalize on the basis of physical stimulus features, and in a manner that produces the peak shift phenomenon.

While the literature demonstrates that humans can generalize in different ways, the question remains as to how these two forms of generalization interact. A study by Livesey and McLaren ([20]) provides one answer. In their study, participants were trained to discriminate between two shades of green differing only in their hue. In two experiments, a peak-shifted gradient was found in the initial phase of testing which gradually became monotonic throughout testing. In Experiment 2, this change in generalization was only found for participants who had failed to notice the difference between the stimuli during training as assessed by a post-experiment interview (those who reported noticing the relation in training produced monotonic gradients from the outset). Presumably, these participants were able to use what they had learned during training to derive the appropriate rule on test. Their within-subjects demonstration of both a monotonic and peak-shifted generalization gradient shows that in the absence of a relational rule, participants generalize according to the physical features of the stimuli.

To summarize, the experimental evidence suggests that the integration of associative and relational information is probably not equal. The fact that peak shift is so elusive suggests that humans predominantly use explicit rules when confronted with discrimination tasks, and if present, this relational rule dominates responding when generalizing at test. This dominance of rule-based responding may be because the majority of human discrimination studies reporting rule-based generalization have used stimuli that are relatively simple with only one relevant dimension (e.g., colored squares, [20]). Although the discriminations are usually difficult enough to ensure that deriving a rule is not easy, once it *has* been derived the application of that rule on test is certainly straightforward, given that participants can identify the rule-relevant difference between the stimuli or categories of stimuli. To date, none of the post-discrimination generalization studies of this nature have observed disruption of the application of rules on test once that rule has been learned.

The aim of the current study was to examine whether reducing the applicability of a relational rule on test could disrupt the application of that rule on trials where the rule should be easy to apply. Rather than restricting the acquisition of relational rules during training, as has been done in the past, the current study provided conditions to encourage the use of a relational rule and then manipulated its applicability on test (that is, the consistency with which the rule can be applied). Our hypothesis was that evidence of feature-based generalization would emerge when the applicability of a rule was reduced at test. The results of Livesey and McLaren ([20]) show that participants’ generalization gradients progress from being peak-shifted towards being monotonic through the course of testing only if participants derive a rule at test and not training, suggesting that in the absence of a relational rule, participants learn about, and generalize according to the physical features of the stimuli. Thus, if knowledge of relational rules and stimulus features are stored concurrently and expression of rule-based generalization dominates under situations where the rule is easy to apply, then reducing the applicability of a rule on test may result in participants reverting to generalizing on the basis of similarity to stimulus features.

Another aim of the study was to provide stronger evidence for rule use by including an additional measure of stimulus similarity. Livesey and McLaren ([20]) found a strong concordance between monotonicity in generalization gradients and verbal description of the relevant stimulus relation (see also [29]). Evidence of rule use at test corresponded with participants reporting that they had noticed the relevant dimension during training. However, this type of analysis inevitably requires post-hoc comparisons from which the relationship between patterns of generalization and articulated knowledge is never completely clear. Furthermore, associative models (e.g., [14]) can also explain monotonic gradients of generalization through feature-based processes, by assuming very broad underlying generalization functions. Under these parameters, the hypothetical peak of the gradient lies close to or beyond the bounds of the test range and thus declining response accuracy would not be observed ([20]). This explanation implies that a monotonic gradient results because participants over-generalize to stimuli that they perceive to be physically similar despite the large differences in at least one physical property. This stands in contrast to rule use, in which a major advantage is that it allows for greater extrapolation of learning to novel situations that are not perceived to be physically similar, that is, instances with low surface similarity but similar structural or relational features ([2,30]).

A simple way to determine if monotonic generalization is based on stimulus properties other than mere physical similarity is to test whether participants simultaneously acknowledge that the stimuli at the extreme ends of the dimension (which should show the highest levels of accuracy) are not similar to the stimuli they have seen during training. Therefore, in addition to category judgements on test, participants were also asked to give typicality ratings as a measure of perceptual similarity. If the presence of a monotonic gradient were due to overly broad generalization and under-assessment of the full range of the dimension, then we would expect typicality ratings to be relatively flat and high across the dimension. On the other hand, if participants are using a relational rule, then categorization accuracy and ratings of typicality should diverge as the test stimuli become less similar to the trained stimuli, with a sharp decline in typicality ratings at the extreme end of the dimension. In summary, the current study aims to investigate whether peak shift can be found in the presence of a relational rule by disrupting rule application, rather than hindering rule formation ([22,31]) or assessing generalization prior to rule formation ([20]) as previous studies have done. In addition, by including typicality ratings, a monotonic gradient of generalization can be more strongly inferred to be the product of relational rule use.

### Experiments

**Fig 2:**
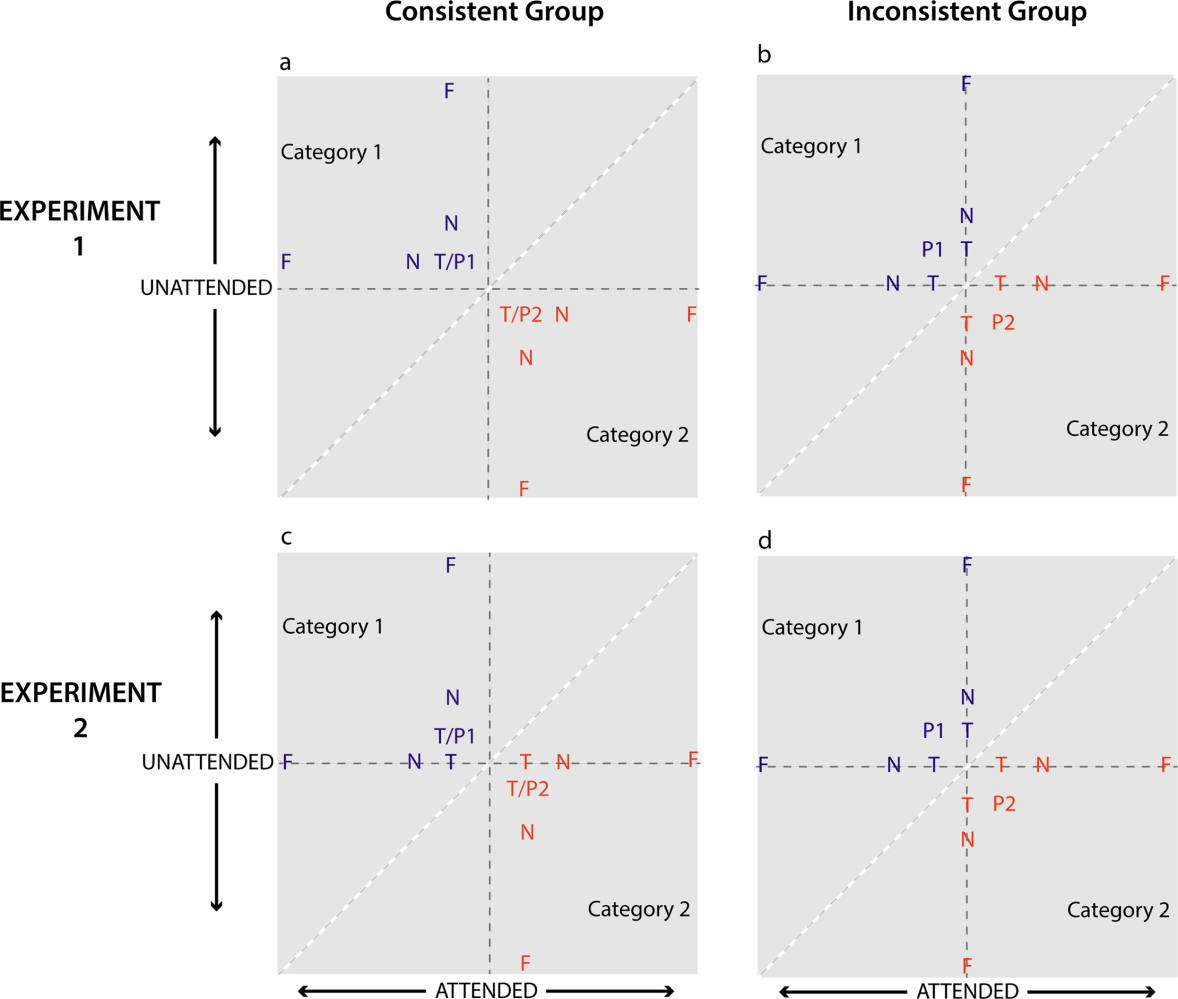
Schematic diagram of the test stimuli in a two-dimensional space for the consistent and inconsistent groups for each experiment. “P” represents the category prototype whose color and size values were seen during training. “T” represents the Train test stimulus which had the same color and/or size values as the training stimuli. “F” represents the Far test stimulus which contains the greenest/bluest or smallest/largest circles. “N” represents the Near1 stimulus. The diagonal white dotted line indicates the category boundary. In Experiment 1, diagnostic information was always present on both dimensions in the Consistent group, but only present on one dimension for any given test stimulus in the Inconsistent group. In Experiment 2, the critical test stimuli (where the attended dimension was varied) were equated between the Consistent and Inconsistent groups. Note that in both experiments, a relational rule using the attended dimension can be used on 100% of test trials in the Consistent group, but only 50% of test trials in the Inconsistent group.

Experiments 1 and 2 examined the effect of disrupting rule application on test by comparing generalization between a Consistent group and an Inconsistent group. In Group Consistent, any relational rules derived during training continued to be applicable throughout the entire test phase. To reduce the applicability of the rule on test in group Inconsistent, we provided a situation where a rule was easily applicable, or useful in determining category membership on 50% of test trials but not easily applicable (and potentially not useful at all) in determining category membership on the other 50% of test trials. In order to prevent participants from simply discounting the trials where the rule was difficult to use as being irrelevant, the test stimuli were created to be perceptually similar to the training stimuli so that participants would assume that their rule would continue to be valid throughout test. We chose stimuli that had two relevant category dimensions and therefore two possible relational rules that participants could form. This allowed us to disrupt rule use by making one dimension (and rule) relevant on half of the test trials, and the other dimension (and rule) relevant on the other half of test trials. We made the stimuli complex with multiple features (9 colored circles on a black background) in order for rule application on test to be more difficult than previous studies, with the hue (blue vs. green) and size of the circles (small vs. large) chosen as the diagnostic category dimensions. The complexity of the stimuli was assumed to make it difficult for participants to determine with confidence whether their rule was applicable on a particular trial, thereby facilitating an effect of our test manipulation. These dimensions were correlated during training, meaning that for example, one category had bluer and larger circles and the other had greener and smaller circles (see stimuli labeled ‘P1’ and ‘P2’ in Fig 2). In order to provide the best opportunity to obtain peak shift, we made the training stimuli perceptually similar and added noise such that relational differences for any given circle were less reliable than the average of all the circles (see S1 Supplemental Materials for full details). This makes the discrimination more difficult but also discourages participants from attending to a single circle, which might have made rule application straightforward and undermine the effect of our manipulation.

**Fig 3:**
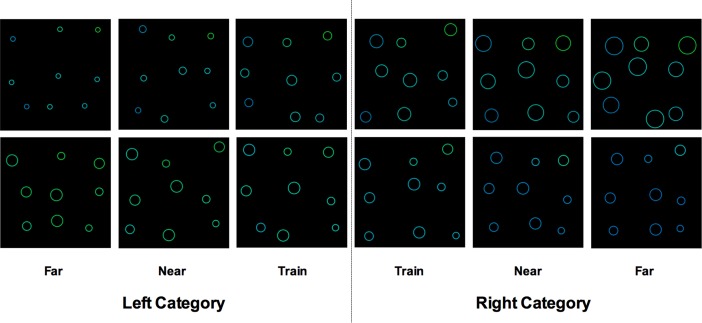
Example stimuli. Examples of a subset of the test stimuli (Train, Near2, Far) seen by the Inconsistent group varying size (top row) and color (bottom row). The left category contains smaller and greener circles, and the right category contains larger and bluer circles. Note how the values of the non-varied dimension (color in the top row and size in the bottom row) do not change between categories. The values of the non-varied dimension were set at a value roughly in the middle of the two categories, making it non-diagnostic of category membership in the Inconsistent group. The Consistent group had diagnostic information from the non-varied dimension, such that there would be a small difference between categories in terms of color in the top row (the same difference present between the Train stimuli in the bottom row), and a small difference between categories in terms of size in the bottom row (the same difference present between the Train stimuli in the top row).

To create the test stimuli, we varied one dimension at a time such that when one dimension was being varied, the other stayed constant. Whether the information on this non-varied dimension was diagnostic of category membership (i.e. varied reliably between categories) was manipulated between groups (see Fig 2). For example, when the color dimension was varied in Group Consistent, the other dimension (i.e. size) was set at the same values used for the training stimuli such that size was still diagnostic of category membership (Fig 2A), but in group Inconsistent, size was set at a value roughly in the middle of the two categories, making it non-diagnostic of category membership (Fig 2B). Fig 3 shows an example of test stimuli seen by the Inconsistent group. Notice that when size was varied (top row), the color values do not change between categories and when color was varied (bottom row), the size values of the circles do not change (but the location of the circles change randomly, providing some noise in the stimuli). This means that if participants were to derive a rule using color, for example, this rule would be difficult to use on test trials when size is varied (Fig 3, top row), and is thus only useful on the half of test trials where color is being varied (Fig 3, bottom row).

In order to increase reliance on a rule at training, and therefore increase the probability of observing an effect of reducing rule applicability at test, participants were explicitly directed to attend to one of the two dimensions (see S1 Supplemental Materials for details of an experiment without this attention manipulation), creating an attended and an unattended dimension (either the color or size of the circles served as the attended dimension, counterbalanced across participants). The focus was on how participants generalized on the attended dimension since this was the dimension where rule use was most likely to occur, and therefore the dimension on which rule disruption was likely to occur. However, generalization along the unattended dimension was also assessed to see whether any learning occurred in the absence of instructed attention. If participants derived a rule using one of the two dimensions, this rule would either be easy to apply on 100% (Group Consistent) or 50% of test trials (Group Inconsistent). Thus, Group Inconsistent would experience a sequence of test trials where their rule was easy to apply on some trials, and very difficult to apply on the other trials, making application of a rule inconsistent. The question of interest was whether manipulating the consistency of rule application on test in this way would affect application of the rule on the test trials where the rule *was* clearly valid and easy to apply.

Experiments 1 and 2 were identical except for a minor procedural detail designed to rule out an alternative explanation of any obtained group differences. In Experiment 1, the Consistent and Inconsistent groups differ not just in the ease of application of rules on test, but also in the slight physical characteristics of the critical test stimuli. As depicted in Fig 2, the Consistent group have diagnostic information present on both dimensions while the Inconsistent group have diagnostic information from a single dimension. Thus, considering the critical ‘attended dimension’ trials, the Consistent group have ‘extra’ diagnostic information on test, which may aid rule use. For instance, for a participant in the Consistent group attending to color, the attended dimension stimuli that vary in color also contain a subtle difference in size that is diagnostic of category membership. Having diagnostic information available during test makes the testing context more similar to the training context than in the Inconsistent group. This is particularly important because despite the proposal that rule use should not be affected by the similarity between training and target domains ([30]), research in different paradigms has shown that in practice, rule use *is* affected by the similarity between the training and test stimuli even when the rule is perfectly valid (e.g., [32,33,34]) and even when using similarity is detrimental to performance ([34]). Consequently, to rule out this alternative explanation for any potential group differences, in Experiment 2, the critical test trials varying the attended dimension were equated between Consistent and Inconsistent groups such that neither group had information on the unattended dimension when the attended dimension was varied (see Fig 2C and 2D). Note that across both experiments, the Consistent group would be able to use a relational rule on their attended dimension on all test trials while the Inconsistent group would only be able to use a rule on their attended dimension on half of the test trials. Due to the high degree of similarity between the designs of these experiments, the data were analyzed together.

## Method

### Participants

One hundred and thirty-nine (*M* age = 20.88, *SD* = 6.22, 96 females) and one hundred and forty (*M* age = 19.48, *SD* = 3.17, 92 females) University of Sydney students participated in Experiment 1 and 2 respectively in exchange for partial course credit or payment (Experiment 1 only, AUD$15/hour). Participants were randomly allocated to either the Consistent (*n* = 134) or Inconsistent (*n* = 145) group. Participants who indicated that they were colorblind were excluded (6 participants).

### Apparatus

The experiments were programmed using Matlab software and PsychToolbox ([35,36]) and run using Apple Mac Mini desktop computers connected to 23 inch Dell monitors and a standard Apple keyboard and mouse. Testing was conducted in groups of up to four and participants were asked to wear headphones to attenuate background noise.

### Stimuli

Each stimulus presented in training and test consisted of 9 circles on a black 600x600 pixel square background. The stimulus background was divided into a 3x3 grid, with each circle confined to a 200x200 pixel cell so that there was no overlap between circles. The location of the circles within each cell varied randomly such that circle location was not predictive of category membership. The critical dimensions that were varied between categories were the color and size of the circles. The minimum and maximum size values (circle radii) were 15 and 50 pixels respectively, and the minimum and maximum color values (hue) were .403 and .555, with saturation and brightness set to 100% and 75% respectively. To allow an adequate assessment of generalization along each dimension, the test stimuli varied between this full range, but the training stimuli were restricted to vary in a mid-range band that included the middle 52% of values. This was done to ensure that the test stimuli were more extreme than the training stimuli to allow an adequate assessment of generalization along each dimension. An example of how stimuli varied along each dimension can be seen in Fig 3, and the full details of how the stimuli were constructed can be found in S1 Supplemental Materials.

### Procedure

Experiments 1 and 2 consisted of three phases, an initial training phase, a categorization test, and a short questionnaire. All instructions and phases were presented within the computer program. Participants gave informed written consent and the experiments were approved by the University of Sydney Human Research Ethics Committee. Participants were randomly allocated to either the Consistent or Inconsistent group, and to either the Attend Color or Attend Size group. The experiment was thus a 2x2x2 between-subjects design with attention group (Attend Color or Attend Size), test group (Consistent vs. Inconsistent), and experiment (1 vs. 2) as the independent variables.

#### Training phase

For each participant, the size and color of the categories were randomized independently such that one category would have greener and the other bluer circles, and one category would have larger and the other smaller circles. Participants first read a cover story explaining that they would see various artworks that belonged to different artists (Evan and Justin), and their task was to work out which artworks belonged to which artist through trial and error. They were warned that the artworks were very similar and thus they might find the task difficult at first, but they would be able to learn about the artworks with feedback. The instructional manipulation was presented on a separate screen prior to the start of the training phase and read:

“*You should try to use as much information about the circles to categorize the stimuli. However, the [COLOUR/SIZE] of the circles may be most helpful in distinguishing Evan’s and Justin’s artworks. Try and attend to the [COLOUR/SIZE] of the circles when learning the categories*.”

Before training started, a message appeared for 3s that told participants to get ready for the task by placing their fingers on the left and right shift keys. On each trial, participants were presented with an artwork in the middle of the screen and the names of the artists appeared on the sides of the screen (“Evan” on the left and “Justin” on the right). Participants made a categorization response by pressing the left or right shift key, and had 4s to respond before they were timed out. Once a response was made or the timeout was reached, the stimulus disappeared, and feedback (either “correct” in black, “wrong” in red, or “too slow” in red) was shown in the center of the screen, along with the choice of artist they had made on that trial. Feedback was shown for 500ms and 1000ms later the next trial would begin. There were 120 trials in total (with 120 unique stimuli, see S1 Supplemental Materials), and the training phase progressed in exactly the same way for both groups.

#### Categorization test

The instructions for the test phase emphasized that participants would now be shown more artworks that belonged to the same artists, and that they should choose which artist the artwork belonged to by again pressing the left and right shift keys, and then make a typicality rating on a scale using the mouse. They were also told that some of the artworks might look like the artworks seen previously, while others would not. It was emphasized in the instructions that the category judgements and typicality ratings were separate judgements, by telling participants that it was possible to know the correct category due to high similarity (i.e. give a high rating for typicality) between a test stimulus and the trained stimuli, or despite a lack of similarity between the test stimulus and training stimuli (i.e. give a low rating for typicality).

The test stimuli consisted of the training stimulus (Train), and transfer stimuli that were more extreme along the dimension (Near1, Near2, Near3 and Far stimuli) for each of the two categories, and for each of the two dimensions. These 20 different test stimuli were presented 4 times each, with the location of the circles randomized each time, such that each instantiation of each stimulus was unique (but the critical color and size values were the same). The only difference between Experiment 1 and Experiment 2 was that the Consistent group had diagnostic information on both attended and unattended dimensions in Experiment 1, but only ever on the attended dimension in Experiment 2 (compare Fig 2A and 2C).

On each test trial, a stimulus was presented with the question “Who does this artwork belong to?” appearing underneath. Participants had unlimited time to respond and pressed the left or right shift key to make their decision. Once they had made their choice a new question replaced the previous one asking “How typical of [chosen artist]’s art collection is this artwork?” and a rating scale appeared underneath the question, ranging from “NOT typical” to “VERY typical”. Participants were free to alter their category choice and typicality rating until they were satisfied with both, after which they could press spacebar to progress to the next trial. There was a blank inter-trial interval (ITI) of 1000ms.

#### Questionnaire

Participants answered a series of questions following the categorization test. The first question asked participants how useful they found various dimensions (brightness, size, color, location) of the circles. Four visual analogue scales appeared on the same page (one for each dimension) and participants made a rating on each scale ranging from “Not useful” to “Very useful”. They could make their four ratings in any order and could only progress to the next question once all ratings had been made. We then asked participants to answer a three-alternative forced-choice question (3AFC) and then a two-alternative forced-choice (2AFC) question about participants’ attended dimension first, and then their unattended dimension. The first 3AFC question read:

“*You may have noticed a difference between the left (Evan) and right (Justin) categories in terms of the COLOUR/SIZE of the circles. If you did notice a difference, when did you notice it?*”

1. During the FIRST phase of the experiment (where there was feedback)
2. *During the SECOND phase of the experiment (where there was no feedback*, *and you had to give typicality ratings)*
3. I did not notice a difference

After answering the 3AFC question, participants proceeded to the 2AFC question, which read:

“*One of the categories had mostly BLUER/LARGER circles, while the other category had mostly GREENER/SMALLER circles. Which of the following do you think is correct?*”

1. *LEFT category (Evan) had mostly GREENER/LARGER circles*, *RIGHT category (Justin) had mostly BLUER/SMALLER circles*.
2. *LEFT category (Evan) had mostly BLUER/SMALLER circles*, *RIGHT category (Justin) had mostly GREENER/LARGER circles*

Both the 3AFC self-report question and the 2AFC rule-identification question were answered by pressing the corresponding number key on the keyboard. Participants also made a confidence rating for the 2AFC rule-identification question on a scale ranging from “I’m guessing” to “100% confident”. All scale ratings were transformed to a scale ranging from 0–100. A manipulation check was also performed asking participants “Which dimension were you asked to attend to? Press 1 for colour or 2 for size”. Lastly, we asked participants to indicate whether they suffered from any form of color-blindness.

## Results and discussion

### Data analysis

The training and test data were analyzed in hierarchical mixed effects regressions using the ‘lme4’ package ([37]) in R ([38]). In the context of our experiments, hierarchical linear regression is more appropriate and powerful than traditional analysis of variance (ANOVA) (see [39]). Firstly, both our training and test data consist of multiple trial-level observations for each level of each factor. Hierarchical linear models utilize the raw trial-level responses (e.g., correct/incorrect), minimizing information loss ([40]). Mixed effects models are especially useful in analyzing categorization test data consisting of binary (correct/incorrect) responses, which otherwise would need to be averaged to form continuous data to analyze in a repeated-measures ANOVA (see [39], for other arguments against using repeated-measures ANOVA to analyze generalization gradients).

In our analyses, we treated subject and trends over test stimuli as random effects, allowing the model to account for subject-level variance by estimating a unique intercept (i.e. categorization accuracy or typicality ratings for the ‘Train’ test stimulus) and slope (i.e. slope of the generalization gradient) for each participant (see [40], for arguments for including both subject and stimuli as random factors). In this way, mixed effects models give more accurate estimates of group effects than traditional ANOVA, as individual differences in intercept and trend are estimated separately to fixed effects in the mixed effects model ([40,41]). For the categorization test data, the presence of linear and quadratic trends was tested in separate models since these trends capture linearity and curvature suggestive of monotonic and peak-shifted gradients respectively. For all other analyses, stimulus was treated as a linear predictor. For brevity, we report only the results concerning the statistical significance of each predictor, using type III Wald Chisquare tests. Note that any significant predictors of categorization accuracy should be interpreted as a change in log odds of responding correctly.

### Exclusion criteria

To ensure that the data we analyzed were from participants who used their hint to derive the appropriate rule, we excluded participants who reported not noticing a difference in their attended dimension during training (58 participants, 21.2% of the sample). It was assumed that despite the difficulty of the discrimination, giving participants a hint should have made it easy to eventually discover a difference between the categories in their attended dimension during training. Thus, any participants who failed this criterion were probably not following the instructions. Participants who failed the manipulation check were also excluded (a further 17 participants, 7.9% of the remaining sample) for the same reason. Note that neither of these exclusion criteria require participants getting the 2AFC rule-identification question correct. We also excluded participants if they did not score > 55% accuracy in the last half of training (a further 14 participants, 6.9% of the remaining sample, see S1 Supplemental Materials for scatterplots of training and test accuracy). After applying these criteria, 184 participants remained (95 in the Consistent group and 89 in the Inconsistent group).

### Questionnaire

**Fig 4:**
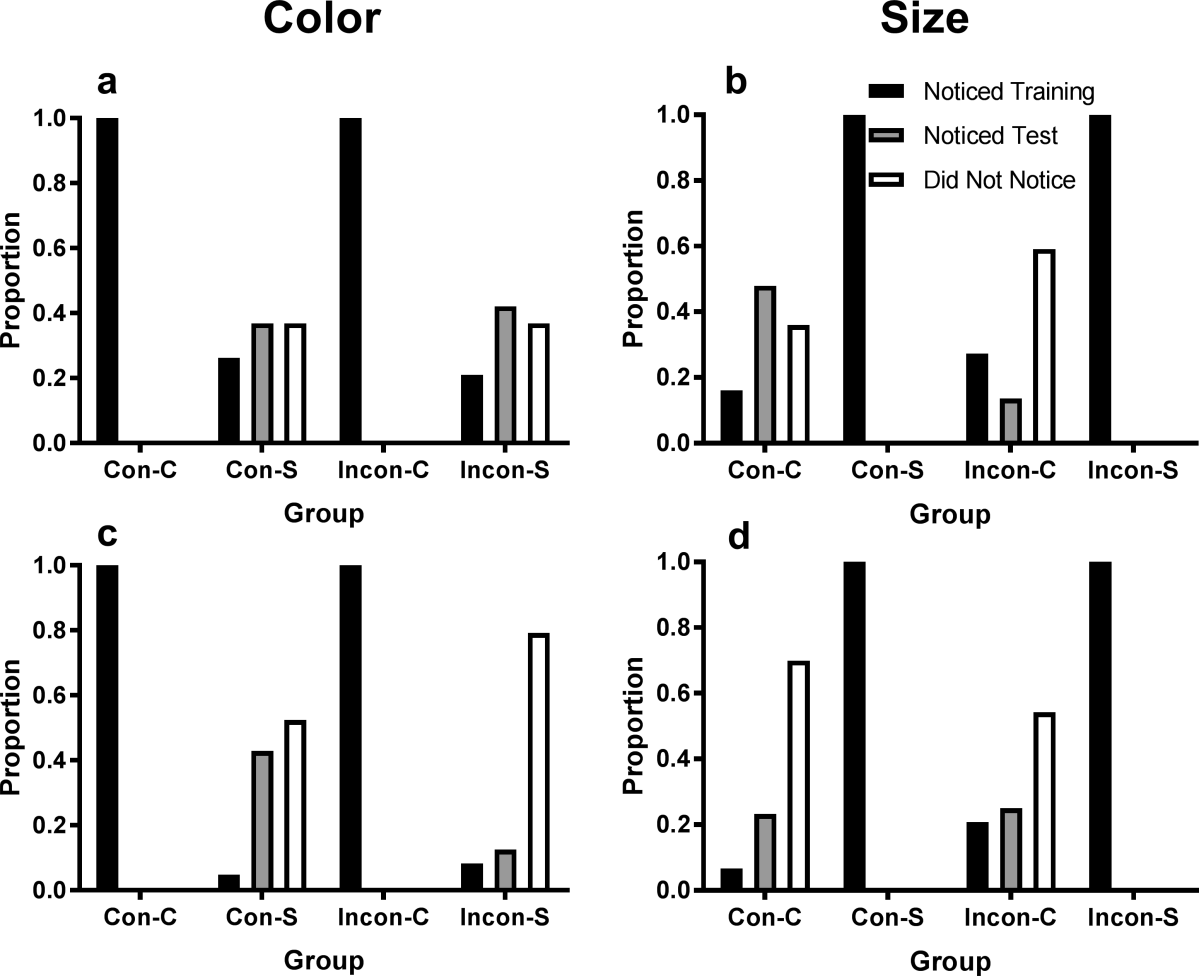
Phase in which category relation was noticed. Proportion of participants who selected each option for the 3AFC self-report question (after exclusions) for each dimension (left panels: color, right panels: size) in Experiment 1 (A and B) and Experiment 2 (C and D). Note that participants who reported not noticing differences in their attended dimension during training were excluded.

**Fig 5:**
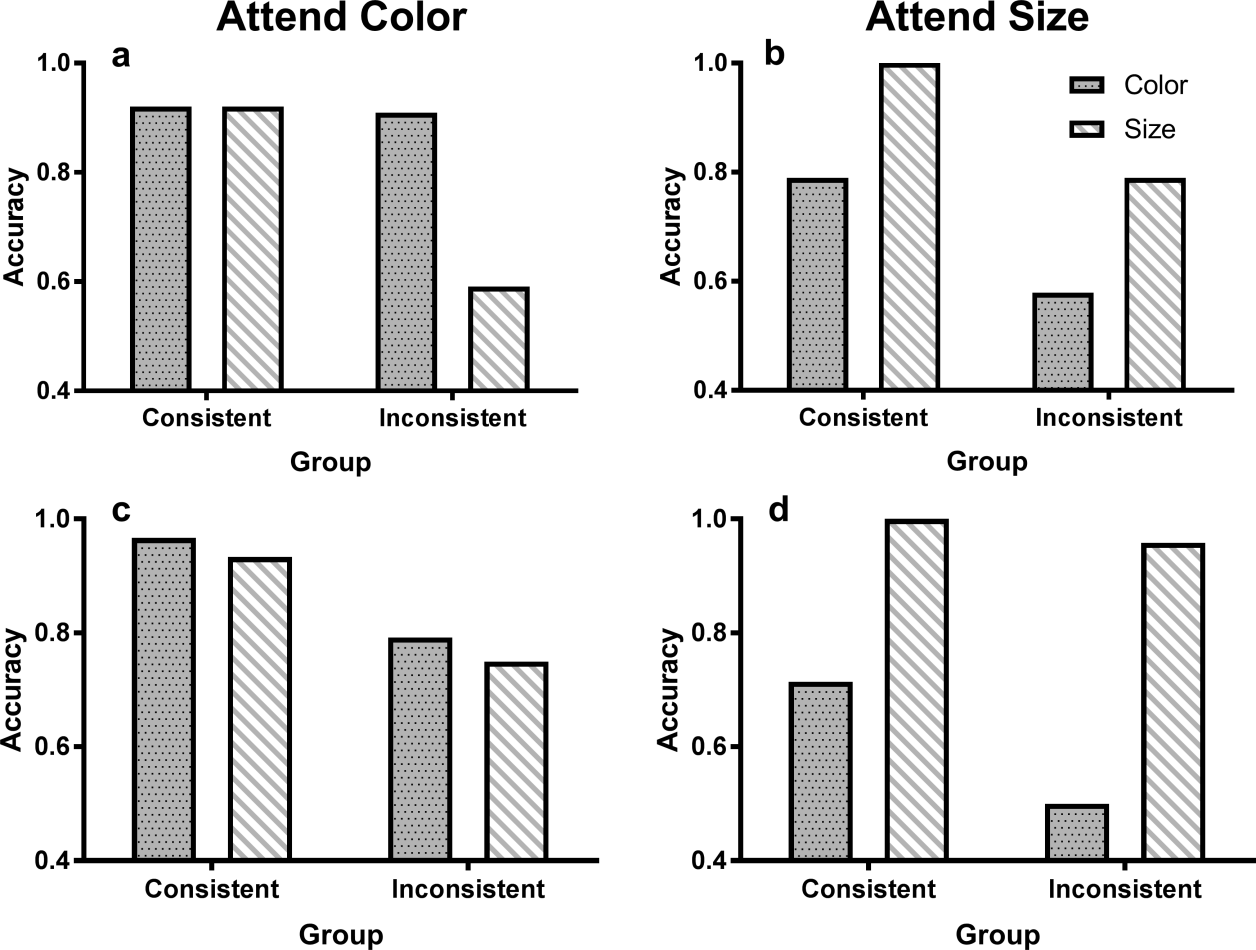
Rule identification accuracy. Accuracy for the 2AFC rule-identification question (after exclusions) for Experiment 1 (A and B) and Experiment 2 (C and D).

Fig 4 shows the proportion of participants in each of the four groups that selected each option in the 3AFC self-report question for color and size in Experiments 1 (4A and 4B) and 2 (4c and 4d) after exclusions. Fig 5 shows the proportion of participants in each group who were able to identify the correct difference between the categories after applying these exclusion criteria in Experiment 1 (5A and 5B) and Experiment 2 (5C and 5D). Accuracy for the rule-identification is generally quite high, and not surprisingly there was significantly higher accuracy for the color dimension when participants were told to attend to color, χ^2^(1, *N* = 184) = 16.9, *p* < .001, (Fig 5A and 5C), and vice versa for the size dimension, *χ*^2^(1, *N* = 184) = 5.49, *p* = .019 (Fig 5B and 5D). The proportion of participants identifying the rule for color and size did not differ between experiments, largest *χ*^2^(1, *N* = 184) = 2.25, *p* = .134, but a larger proportion of participants in the Consistent group were able to identify the correct category difference for color, *χ*^2^(1, *N* = 184) = 6.54, *p* = .011, and also for size, *χ*^2^(1, *N* = 184) = 11.9, *p* < .001. A possible explanation for these group differences in rule identification is that the reduction of rule applicability at test may have resulted in more errors when attempting to identify the rule in the subsequent questionnaire.

### Training

The training data were analyzed in a logistic hierarchical regression with test group (Consistent vs. Inconsistent), attention group (Attend Color vs. Attend Size), experiment (1 vs. 2), and training block (1–4) as predictors, and estimating a random intercept and slope for each participant. Training block predicted linear increases in accuracy, *χ*^2^(1, *N* = 184) = 11.7, *p* < .001, and did not interact with test group, *χ*^2^(1, *N* = 184) = 1.07, *p* = .300, nor attention group, *χ*^2^ < 1, but did interact with experiment, *χ*^2^(1, *N* = 184) = 6.23, *p* = .013. This interaction was due to steeper learning curves in Experiment 2. Due to these training results, the following analyses will collapse over attention group and experiment but we also ran an additional model for each analysis including experiment as an additional predictor. We have noted where any results interacted with experiment.

### Category judgements

**Fig 6:**
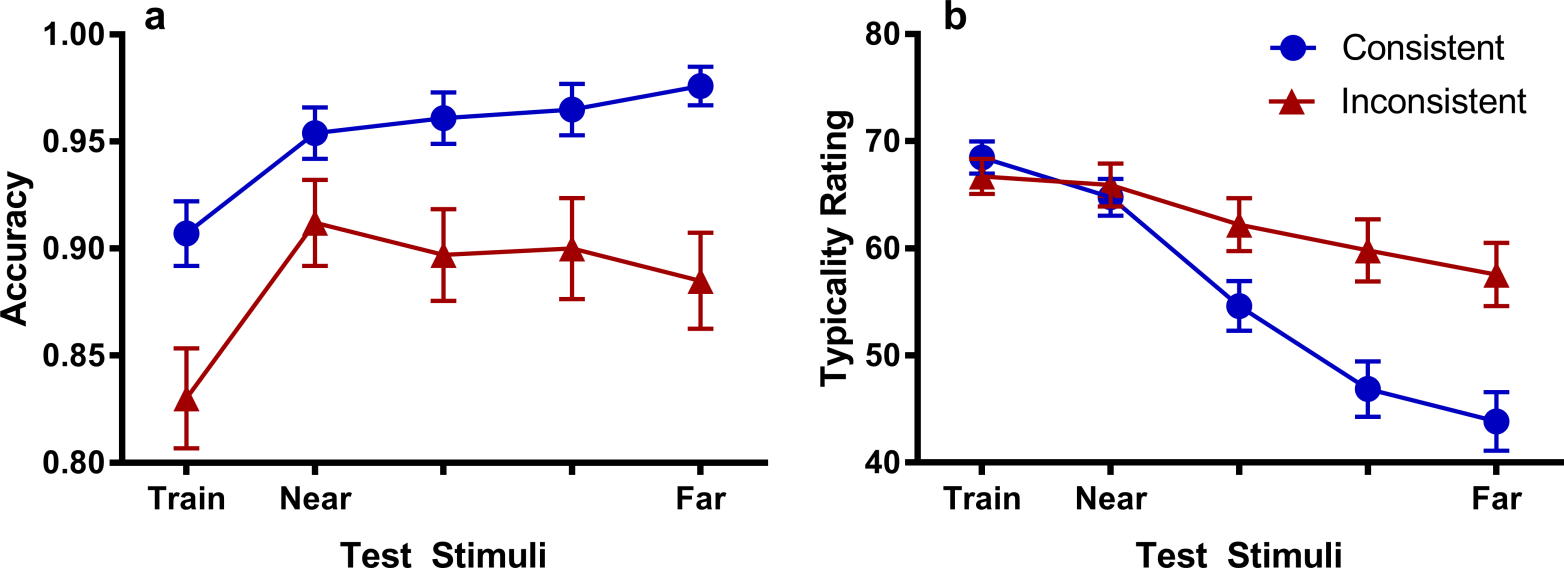
Generalization gradients for the attended dimension. Categorization accuracy (A) and typicality ratings (B) for test stimuli varying the attended dimension in Experiments 1/2. Error bars represent the standard error of the mean. Note that the scales on the y-axis do not start at 0.

The categorization results for variations along the attended dimension are shown in Fig 6A. Inspecting Fig 6A, a monotonic gradient seems to be present in the Consistent Group, while the Inconsistent Group is clearly showing a different pattern of generalization that resembles a peak shift. We fitted a logistic hierarchical mixed effects model to the trial-level binary (correct/incorrect) data including stimulus as a linear predictor, group as a nominal predictor, and estimating a random intercept and slope (across the stimulus predictor) for each participant. There was a significant effect of group, *χ*^2^(1, *N* = 184) = 4.25, *p* = .039, meaning that the Consistent group were significantly more accurate in categorizing the test stimuli than the Inconsistent group. Stimulus was found to be a significant linear predictor of the trend in categorization accuracy, *χ*^2^(1, *N* = 184) = 41.7, *p* < .001, and there was a significant interaction between stimulus and test group, *χ*^2^(1, *N* = 184) = 7.06, *p* = .008. The results were similar for the model testing for a quadratic trend over the stimulus dimension, with test group, *χ*^2^(1, *N* = 184) = 5.07, *p* = .024, stimulus, *χ*^2^(1, *N* = 184) = 26.6, *p* < .001, and their interaction, *χ*^2^(1, *N* = 184) = 8.40, *p* = .004, all significant. Therefore, it appears that reducing rule applicability at test changed the shape of the generalization gradient.

Since we obtained significant interactions between stimulus and group, we conducted planned simple effect analyses testing for linear and quadratic trends within each group and a peak shift in the Inconsistent group. In both groups, stimulus was found to be a significant linear, and quadratic predictor of categorization accuracy, *χ*^2^s(1, *N* = 89) > = 9.98, *p* = .002. Thus, it appears that linear and quadratic trends in categorization accuracy were present in both groups, but the linear trend was stronger in the Consistent group, and the quadratic trend was stronger in the Inconsistent group. To test for the presence of peak shift in the Inconsistent group, we compared the highest of the three middle points (Near1, Near2, Near3) to the two endpoints (Train and Far) in a logistic hierarchical regression. These tests are necessarily one-tailed since peak shift involves a significant rise and fall in accuracy. Since we tested the highest of the three middle points, the critical Bonferroni-corrected alpha value used for both tests was .033. Using the Near1 point, there was a significant rise in accuracy from the Train stimulus, *χ*^2^(1, *N* = 89) = 27.4, *p* < .001, and a marginally non-significant fall in accuracy to the Far stimulus using the Bonferroni-corrected alpha value of .033, *χ*^2^(1, *N* = 89) = 4.33, *p* = .037. Although the statistical evidence for the presence of the peak-shifted gradient in the Inconsistent group was weak, differences in the shape of the generalization gradient and overall poorer performance in the Inconsistent group relative to the Consistent suggest that rule-based generalization was disrupted in the Inconsistent group.

**Fig 7:**
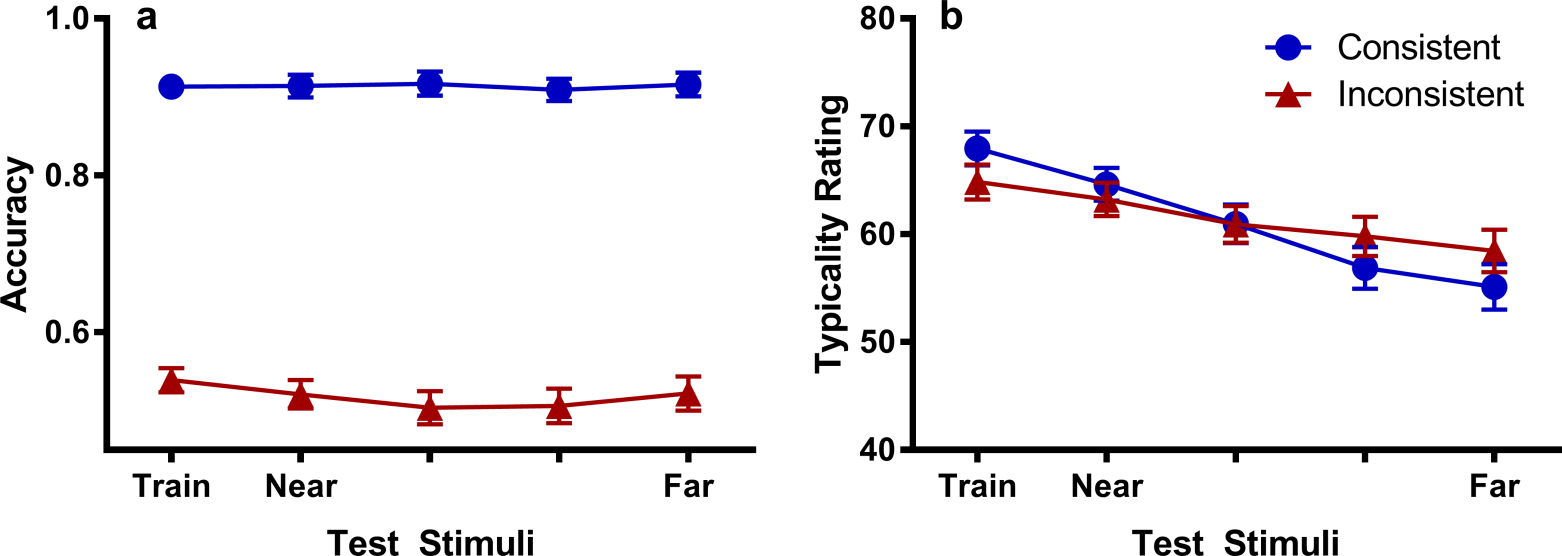
Generalization gradients for the unattended dimension. Categorization accuracy (A) and typicality ratings (B) for test stimuli varying the unattended dimension in Experiments 1/2. The data in Experiments 1 and 2 were combined since there were no significant differences found between experiments. Error bars represent the standard error of the mean. Note that the scales on the y-axis do not start at 0.

Categorization accuracy for generalization along the unattended dimension is shown in Fig 7A. The data were analyzed in a similar way to the attended dimension, but treating stimulus as a predictor of linear trend only. Stimulus was not found to be a significant linear predictor, *χ*^2^(1, *N* = 184) = 2.85, *p* = .091, and stimulus did not interact with test group, *χ*^2^(1, *N* = 184) = 3.30, *p* = .069, although this effect was marginal, and probably due to the slight linear trend evident in the Inconsistent group (Fig 7A). However, the Consistent group had significantly higher accuracy than the Inconsistent group overall, *χ*^2^(1, *N* = 184) = 191.5, *p* < .001. The high level of accuracy attained by the Consistent group can be explained by the fact that on these trials, information on the other (i.e. attended) dimension was present, and participants must have relied on this dimension for their category judgements. The Inconsistent group on the other hand, did not have information on the other (attended) dimension and accuracy is therefore close to chance. The flat generalization gradients in each group indicate that the unattended dimension did not acquire any stimulus control.

### Typicality ratings

The typicality ratings for the attended dimension are shown in Fig 6B. It can be seen that while both groups exhibit a descending linear gradient in their ratings, the gradient for the Consistent group is clearly steeper than the Inconsistent group. These observations were confirmed statistically in that stimulus was found to be a significant predictor of typicality ratings, *χ*^2^(1, *N* = 184) = 103.6, *p* < .001, and interacted with test group, *χ*^2^(1, *N* = 184) = 20.3, *p* < .001. Note that stimulus did interact with experiment, *χ*^2^(1, *N* = 184) = 3.99, *p* = .046, once experiment was added to the model. Group was not found to be a significant predictor, *χ*^2^ < 1. The typicality ratings show that the highest categorization accuracy was achieved for the test stimuli that participants considered to be least typical of the category, providing further evidence that participants in the Consistent group were generalizing on the basis of a relational rule. A monotonic gradient in categorization accuracy can always be argued to indicate the rise of a peak-shifted gradient that spans a large width of the category dimension ([20]), but the presence of these descending typicality gradients confirms that the monotonic gradient in categorization is based on more than just the perceived similarity of the test stimuli to those seen in training.

Similar results were found for typicality ratings on the unattended dimension (Fig 7D). Again, stimulus was a significant predictor of typicality ratings, *χ*^2^(1, *N* = 184) = 73.9, *p* < .001, with significantly steeper gradients in the Consistent group than the Inconsistent group, *χ*^2^(1, *N* = 184) = 9.49, *p* = .002. There was no effect of group, *χ*^2^(1, *N* = 184) = 2.12, *p* = .146.

### Summary

In summary, we found, for category judgements of stimuli varying along the attended dimension, evidence of reduced accuracy in the Inconsistent group, as well as a different pattern of generalization to the Consistent group. Critically, these results suggest that reducing the applicability of a rule on test disrupted rule-based generalization. Neither of these effects interacted with experiment, indicating that they were not due to the subtle additional information conveyed on the unattended dimension in the Consistent condition in Experiment 1. In the Consistent group, a monotonic gradient of generalization was present, consistent with rule use. Although a weaker (though still significant) linear trend in generalization was also present in the Inconsistent group, the gradient appeared to be non-monotonic, peaking at an intermediate stimulus value close to the trained stimuli. However, planned analyses of the peak-shifted gradient revealed that the decline in accuracy to the extremes of the test range was marginally non-significant. Thus, although the shape of the gradient is consistent with generalization on the basis of similarity to physical features, the overall evidence for a peak shift was statistically weak. Evidence for generalization on the basis of a rule was provided by the typicality ratings, where the Consistent group performed at the highest level of accuracy for stimuli they rated as least typical of the category. Interestingly, our test manipulation affected not just category judgements but perceptions of typicality, with the Inconsistent group showing a flatter gradient in their typicality ratings. It is clear that while the Inconsistent group displayed a gradient resembling a peak shift, the decline in accuracy from the peak to the extremes of the test range was statistically weak and the linear trend (indicating, more generally, an increase in accuracy across the dimension) was also significant. This pattern of statistical results suggests that participants were generalizing on the basis of a rule *and* similarity. This is to be expected if participants have derived a rule and are simply unsure whether to use it and therefore show a mixture of generalization gradients. The effect of reducing rule validity might therefore be to undermine certainty or confidence in applying the rule, and it may be the case that this certainty changes on a trial-by-trial basis, with the pattern of generalization dictated by rule applicability on the previous trial.

### Sequential re-analysis

**Fig 8:**
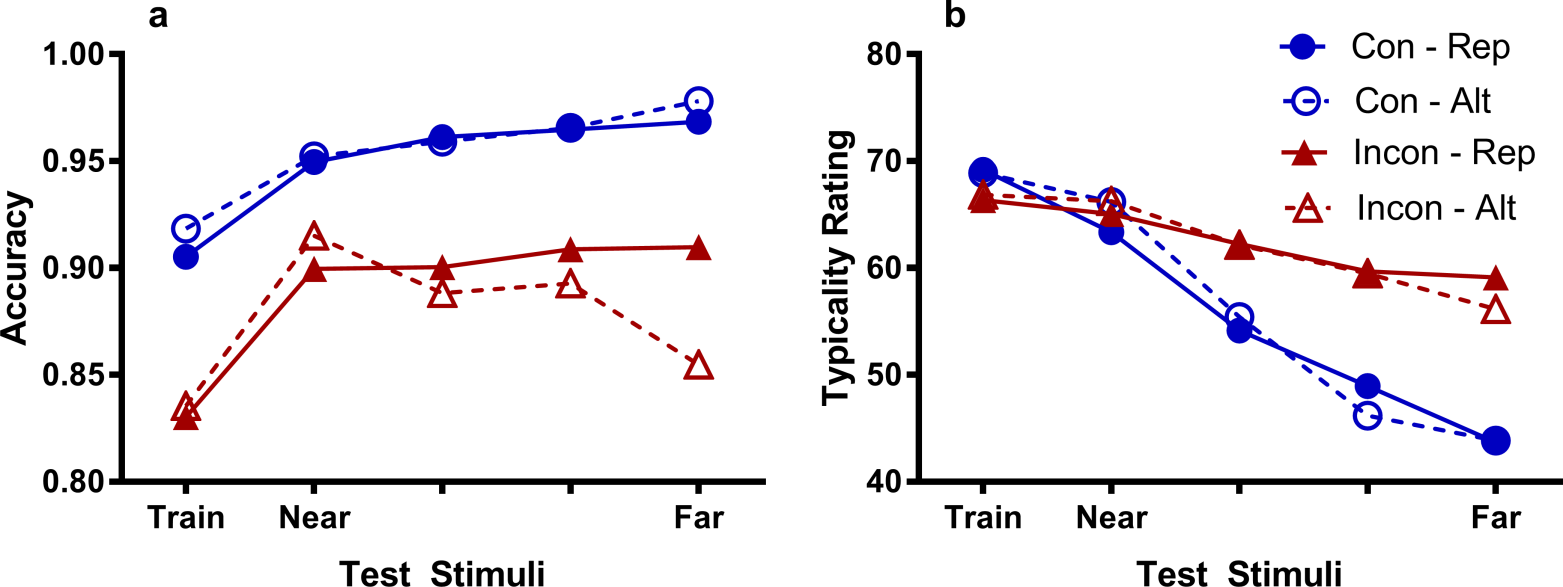
Sequential re-analysis for the attended dimension. Fig shows categorization accuracy (A) and typicality ratings (B) for test stimuli varying the attended dimension. Trials are further divided according to whether the rule on the attended dimension was applicable on the previous trial (i.e. the previous trial varied the same dimension, ‘repetition’ trials) or not applicable on the previous trial (i.e. the previous trial varied the unattended dimension, ‘alternation’ trials). Note that the scales on the y-axis do not start at 0.

To test this hypothesis, we conducted a post-hoc sequential analysis of the categorization data in both experiments whereby the “attended dimension” test trials were divided into “repetition” and “alternation” trials. Repetition refers to trials where the previous trial varied the same (i.e. attended) dimension (and therefore a rule on the attended dimension *was* previously applicable in both groups), and alternation refers to trials where the previous trial varied the other (i.e. unattended) dimension (and therefore a rule on the attended dimension *was not* previously applicable in the Inconsistent group but *was* applicable in the Consistent group). The results for the sequential analysis are shown in Fig 8A, with the corresponding typicality ratings shown in Fig 8B. It is firstly apparent that the Consistent groups are showing roughly monotonic gradients for both repetition and alternation trials, but the Inconsistent groups are showing different patterns of generalization based on the previous trial. Due to this being a post-hoc analysis, only the results of interest will be reported concerning categorization accuracy.

#### Inconsistent group

The data for the Inconsistent group were analyzed in a logistic hierarchical regression with stimulus as a continuous predictor, previous trial (repetition vs. alternation) as a nominal predictor, and including a random intercept and slope for each participant. As before, two separate models were fitted, one treating stimulus as a linear predictor and another treating stimulus as a quadratic predictor. Stimulus was found to be a significant predictor of linear, *χ*^2^(1, *N* = 89) = 13.7, *p* < .001, and quadratic, *χ*^2^(1, *N* = 89) = 6.89, *p* = .009, trends in categorization accuracy. Importantly, there was a significant interaction between stimulus and previous trial in the quadratic model, *χ*^2^(1, *N* = 89) = 4.54, *p* = .033, and a marginally non-significant interaction between stimulus and previous trial in the linear model, *χ*^2^(1, *N* = 89) = 3.61, *p* = .057. There was no overall effect of previous trial in either model, largest *χ*^2^ < 1. The significant interaction suggests that the shape of the gradient within the Inconsistent group varied depending on whether the primary rule was applicable or inapplicable on the previous trial. In particular, for the alternation trials, there appears to be a peak-shifted gradient that is not present in the repetition trials. Supporting this observation, using the Near1 point, there was a significant rise in accuracy from the Train stimulus, *χ*^2^(1, *N* = 89) = 30.2, *p* < .001, and a significant fall in accuracy to the Far stimulus, *χ*^2^(1, *N* = 89) = 5.02, *p* = .025, for the alternation trials. Thus, dividing the trials based on the applicability of the rule on the previous trial appears to have strengthened the peak shift effect on the alternation trials, while the gradient for the repetition trials appears to be more linear, like the gradient in the Consistent group. Notably, on these repetition trials the Inconsistent group’s performance still did not match the absolute levels attained by the Consistent group, suggesting a strong overall impairment as a result of the test manipulation. On trials where the previous trial varied the other dimension and the rule could not be used, participants did not consistently generalize using a relational rule but instead generalized on the basis of similarity, showing a peak shift.

#### Consistent group

In contrast, the Consistent group did not show the same interaction between stimulus and previous trial in either the linear, *χ*^2^ < 1, nor the quadratic model, *χ*^2^ < 1, although stimulus was found to be both a significant linear, *χ*^2^(1, *N* = 95) = 23.8, *p* < .001, and quadratic, *χ*^2^(1, *N* = 95) = 14.0, *p* < .001, predictor of accuracy. There was no effect of previous trial in either model, *χ*^2^s < 1. Therefore, whether the same or different dimension was varied on the previous trial had no effect on generalization in the Consistent group, who showed consistent rule-based generalization throughout test, since they could use their rule on every trial. None of these effects interacted with experiment.

Thus, within the same participants in the Inconsistent group, a peak-shifted and monotonic gradient of generalization were both observed, depending on the applicability of a rule on the previous trial. In contrast, the typicality ratings in the Inconsistent group did not appear to differ in the same way (see Fig 8B), suggesting that what was changing on a trial-by-trial basis was participants’ likelihood of using a relational rule based on its recent applicability, rather than perceptions of similarity between the test and training stimuli. This analysis reinforces our conclusion that disrupting rule use serves to undermine participants’ certainty or proficiency in applying their rule and leads them to switch between generalizing on the basis of rules and similarity on a trial-by-trial basis. The results of this sequential analysis indicate that participants apply rules in a flexible way as a function of recent difficulty in the applicability of the rule, suggesting an important condition that can limit rule use.

Given previous literature demonstrating that peak shift only occurs in the absence of a relational rule (e.g., [20,27]), the evidence suggestive of a peak-shifted gradient in the Inconsistent group is striking since the majority (77/89) of participants in the Inconsistent group *were* able to correctly identify the rule-relevant difference between categories. The fact that we explicitly directed participants to use a particular dimension during training, and the fact that we excluded participants who claimed that they did not notice any difference between the categories during training given this hint, leaves little chance that the remaining participants were mostly guessing the category relation correctly. However, the present result can easily be accommodated by assuming that rule-based generalization only occurs when the rule is both easy to derive *and* apply.

## General discussion

In this study, we compared categorization accuracy and typicality ratings between a group who were able to derive a relational category rule during training and apply that rule consistently on test, and a group who could only use that rule inconsistently. During training, the color and size of stimulus features were predictive of category membership and it appeared that instructing participants to attend to one of the dimensions facilitated derivation of a relational category rule on that attended dimension. On test, participants either experienced test trials where information was present on both color and size dimensions, making a rule on the attended dimension relatively easy to apply on all trials (Consistent group), or test trials where information was only present on the attended dimension on half of the test trials, making a rule about that dimension very difficult to apply on 50% of trials and thereby rendering its application on test inconsistent (Inconsistent group). The test trials of interest were those in which the rule *was* clearly valid and easy to apply in both groups (i.e. where the attended dimension was varied). These test stimuli were slightly different in Experiment 1 but equated in Experiment 2, and these slight physical differences were not found to influence the results. Although participants showed strong evidence of learning and reported noticing simple relational rules, when rule application was made difficult on test, a generalization gradient that appeared peak-shifted emerged, with declining accuracy at the extrema of the test range despite the majority of participants being able to identify the relational category rule. Although the peak shift was statistically weak, the critical group interactions, when integrated with the existing human literature on peak shift, suggest that when formation or *application* of a relational rule is difficult, generalization gradients resemble a peak shift more than a monotonic gradient.

Although our main hypothesis did not find support in the analysis of the overall test data, a posthoc sequential analysis suggested that this was due to participants in the Inconsistent group exhibiting different patterns of generalization depending on rule applicability on the previous trial. Our sequential analysis showed a within-participant dissociation in generalization based on whether the rule was applicable on the previous trial. Rule applicability on the previous trial had no effect on the monotonic gradients displayed in the Consistent group, while the Inconsistent group showed a peak shift on trials where they could not use their primary rule easily on the previous trial, and a monotonic gradient on trials where they could use their rule on the previous trial. This not only confirms that participants in the Inconsistent group did acquire a relational rule but also reinforces our conclusion that reducing rule applicability at test serves to undermine the likelihood of applying that rule. A much stronger peak shift was seen in the sequential analysis than in the overall test data, since the overall test data was composed of trials in which participants generalized in different ways. In contrast, participants in the Consistent group showed a monotonic gradient that was unaffected by the nature of the previous trial, presumably since their rule was applicable on all test trials. The sequential re-analysis suggests that participants can engage in both rule-based and feature-based generalization during test, with the expression of each varying according to the difficulty of applying the rule on the previous trial. This finding is noteworthy as it suggests that the formation of a relational rule is not always overridden by a permanent switch to rule-based generalization, as suggested in Livesey and McLaren ([20]). In other words, our study adds to the previous human peak shift literature in presenting a novel eliciting condition for peak shift that surprisingly, involves the presence of a relational rule. We note however, that this analysis was post hoc, and should be interpreted in light of its exploratory aim.

### Typicality ratings

Interestingly, typicality gradients were found to be steeper for the Consistent group than the Inconsistent group (Fig 6B). The flatter typicality gradient in the attended dimension for the Inconsistent group can be explained if we assume that participants are attending less to their attended dimension than group Consistent. Disrupting rule validity on test may have led participants in group Inconsistent to search for additional information other than their attended dimension to aid their judgements. Since at test it is now obvious that two dimensions are varying, participants may have started focusing on the unattended dimension as well. As these values did not change between stimuli, typicality ratings incorporating information on the unattended dimension would thus be flatter. However, since the same pattern of results was also found for the unattended dimension, a more likely explanation is that participants in the Inconsistent group may have started to attend to the irrelevant features of the stimuli during the test phase (e.g., the location of circles), which would have the same effect on both dimensions. In fact, any attention directed away from the relevant dimension when judging typicality would have made the typicality gradient flatter, and therefore explain the flatter gradient observed in the Inconsistent group.

Further, since the group difference did not interact with experiment, we cannot explain these results using differences in the ‘attended dimension’ stimuli presented to the Consistent and Inconsistent groups on test, since these were equated in Experiment 2. It would seem that when a rule is rendered less valid on test in the Inconsistent group, participants overgeneralize perceptions of typicality to non-typical category members in comparison to the Consistent group, effectively considering dissimilar stimuli as being part of the category. This may reflect uncertainty in the boundaries of the category as a direct result of the lack of applicability of a rule. This is an intriguing result as it suggests that undermining the applicability of a relational rule has effects not just on categorization accuracy, but also when judging perceptions of typicality of novel category members. Whether this was a direct result of disrupting rule use or the result of changes in attention is not determinable in our study, and is a potential avenue for future research.

### Mechanisms responsible for monotonic generalization gradients

Similar to previous authors, we have interpreted monotonic generalization over a wide range of test stimuli to be indicative of the use of a relational rule. However, one problem with assuming that monotonic categorization is synonymous with relational rule use is that the same pattern can be derived from other psychological processes. In principle, an associative model (e.g., [14]) can simulate a monotonic gradient if a broad generalization gradient is assumed which spans a wide range of the dimension ([20]). The monotonic gradient can thus be interpreted as the rise in accuracy in a peak shift that has not been given the opportunity to decrease due to testing a limited range. Our inclusion of typicality ratings addresses this problem to some extent. It is clear that the Consistent group, who show greater evidence of a monotonic gradient, do not show broader generalization on the typicality ratings. In fact, generalization gradients for typicality fell more sharply in this group compared to the Inconsistent group. Our typicality ratings complement the evidence from self-report measures that monotonic gradients are correlated with identification of the underlying relationships between stimuli. This evidence is important because relying on self-report measures to divide and compare participants is effectively correlational and, furthermore, it has been argued that participants have poor introspective skills and can report knowledge of rules that were not responsible for their behavior at test ([42]). The declining typicality ratings for the Consistent group lend some validity to using self-report measures to assess rule use and category knowledge.

### Mechanisms responsible for peak-shifted generalization gradients

It is tempting to conclude that the peak shift shown in the Inconsistent group on trials where the relational rule was not applicable on the previous trial is due to the same basic associative processes used to explain peak shift in animals. Error-correction learning models account for the peak shift phenomenon with impressive quantitative precision ([7,10,14], but see [15], for an alternative rule-based explanation]. When considered in light of the human literature on peak shift, our results seem wholly consistent with the idea that associative processes always operate in human categorization or discrimination studies, but are usually masked by higher-order rule-learning which dominates performance at test ([20]). Relational rules, especially simple ones that are easy to articulate and apply, should reduce the difficulty of discriminating between categories and thus it is to the participants’ advantage to search for a rule to improve their performance. If rules have primacy over associative information in governing responding, then only when the task requirements and stimuli make it difficult to derive a rule (e.g., [22,27]) can evidence of peak shift emerge.

It is also possible that participants in the Inconsistent group are generalizing on the basis of a similarity rule that is closely tied to the physical characteristics of the experienced category. Note that the content of learning in a rule of this form is still clearly different to the content of learning of a relational rule that describes the relationships between the stimuli, and that such a rule would need to explain why performance does not peak at the value of the training stimulus. For example, peak shift can be explained if we assume that participants are adopting a conservative decision-rule (e.g., “respond left when the circles are mostly small, but not too small”) whereby the training stimuli are too similar to each other to enable confident categorization but the stimuli that are slightly more extreme are less likely to suffer this problem. Whatever the exact specification of the similarity rule, the results of the sequential analysis can be explained by participants switching between two rules–one based on similarity and the other based on relations.

Whether this switch is a deliberate decision or more automatic is difficult to determine. It may be that rule use, despite being a higher-order function, exhibits sequential effects just like more simple responses and predictions (e.g., [43,44]). However, instead of explaining these sequential effects through activation or priming of an event representation, the rule itself is primed through its utility on the previous trial. This priming process may be relatively automatic, but would still imply a conscious recognition of the applicability of the rule on each trial since no feedback is given pertaining to the correctness of participants’ answers or the utility of their rule. Participants could also be deliberately switching strategy between trials, or simply ‘trying’ the other category response as a result of uncertainty in their rule. This would certainly account for the lower overall accuracy seen in the Inconsistent group, but does not explain how the monotonic gradient could change to peak-shifted gradient. To explain peak shift, such an account would also need to incorporate some moderating effect of stimulus similarity on the likelihood of trying the alternative category response.

Indeed, one interpretation of peak shift may be that it reflects relational rule use that is modulated by stimulus similarity. Participants may know, for example, that stimuli in the left category had smaller circles, but at the extreme ends of the dimension where the circles become very small, participants may be uncertain of the applicability of their rule due to the stimuli being too dissimilar to the training stimuli. Although possible, this explanation seems unlikely due to the reasonable limits on the range of each dimension tested (see Fig 3). There is no obvious reason why participants would regard their rule as applicable to the Near1 stimuli but not to the Near3 and Far stimuli. Still, the evidence showing that rule use is affected by similarity between test and training stimuli (e.g., [32,34]) suggests that further exploration of the nature of rules that participants derive and their willingness to use that rule on extreme test stimuli is needed. For example, it may be that the degree to which participants show a peak-shifted gradient is related to their degree of certainty in a relational rule.

### Interaction between rules and similarity

Livesey and McLaren ([25]) showed that the emergence of a relational rule during test resulted in a peak-shifted gradient being replaced by a monotonic one, leading them to suggest that the operation of rule learning and other executive processes meant that evidence of associative processes would be hard to find. The current study however, shows that the presence of a relational rule does not entail that the expression of feature-driven learning will be completely overridden. Rather, our study shows that under certain conditions, relational rules can only be fully expressed on trials where participants are confident that it is applicable. This finding is also consistent with Natal, McLaren, and Livesey ([45]) who proposed that the content of associative learning and rules are integrated into category judgements and subject to cognitive control. In their study, they found different levels of accuracy and patterns of generalization based on whether participants could verbalize a relational category rule. However, they found that both rule-learners and feature-learners were able to reverse the category assignments when asked to, suggesting that associative learning in this context does not automatically bias actions and instead is subject to cognitive control. Similarly, the fact that peak shift was found once rule use was disrupted suggests that learning about a relational rule does not preclude learning about physical features, but that feature learning is only expressed under conditions where participants are not willing or able to use a rule. If peak shift is interpreted as the result of associative learning mechanisms, our results are consistent with the idea that associative learning processes operate alongside rule learning, but may not always be expressed due to rule-based generalization having priority on test ([25]).

Our results are also relevant to theories of categorization that propose two separate, qualitatively different learning processes (e.g., [24,46]), as well as hybrid models of categorization. Hybrid models assume that rule- and similarity-based processes are qualitatively similar (e.g., [47,48,49]), but vary based on the number of dimensions considered ([48]), the abstractness of the feature that is considered ([49]) or whether attention is directed to rules or exemplars ([47]). In particular, models that assume competitive processes with only a single process ‘winning’ and determining behavior would need to incorporate an assessment of rule applicability to explain our results. With both dual-process and hybrid models of categorization, the utility of a process is assumed to determine its use ([34]). Thus, our results might be consistent with these models if it is assumed that our test manipulation reduced the utility of using a rule and therefore decreased its ability to influence responding. Further studies investigating other factors that cause participants to switch between using relational rules and similarity may further inform the development of models that assume competitive processes, whether qualitatively distinct or similar.

### Conclusion

In conclusion, our results show that in simple category learning, the presence of a relational rule does not necessarily entail that a monotonic gradient of generalization will result. Rather, whether the test conditions make that rule applicable determines whether participants show a monotonic or a peak-shifted gradient of generalization. When rule application on test was consistent, participants generalized using a relational rule, but when rule application on test was rendered inconsistent, participants may have been uncertain of their rule and reverted to generalizing on the basis of similarity. Importantly, we have provided a within-subjects demonstration of participants switching between rule- and similarity-based generalization as a function of rule applicability on the previous trial, suggesting that both kinds of learning are stored concurrently, and their respective influences on behavior can change dynamically throughout test.

## Supporting information

S1 Supplemental materials (PDF)
