# Supplementary material for: Rule-based generalization and peak shift in the presence of simple relational rules
Source: PLoS One. 2018 Sep 14;13(9):e0203805. doi: 10.1371/journal.pone.0203805 (PMC6157829; doi:10.1371/journal.pone.0203805)
Supplement: S1 Supplemental materials — (PDF) [file pone.0203805.s001.pdf]

Supplemental Materials

Appendix A: Stimulus Construction

All stimuli consisted of 9 circle outlines (5 pixels thick) on a black square background that was 600 x 600 pixels in size. The square background was divided into a 3 x 3 grid with each square measuring 200 x 200 pixels containing each of the 9 circles. For each stimulus in training and test, the location of each circle within its 200 x 200 pixel cell varied randomly, such that the location of the circles was noisy and not predictive of category membership. Restrictions on circle location and size were put in place to ensure that none of the circles overlapped with each other or the square boundary (see Table 1). The circles had two other values that could vary: color (hue) and size (circle radius), and these were the dimensions on which the categories differed. The minimum and maximum circle radii used were 15 and 50 pixels respectively, and the hue varied between .403 (green) and .555 (blue), with saturation and brightness set at 75% and 100% respectively (see Table 1). If any values crossed these boundaries they were simply set at that boundary. The hue values were chosen so that the minimum value was obviously green, the maximum value was obviously blue, and the middle value was ambiguous.

Table A. Stimulus hue and size values.
Min and max values for the whole dimension (Min, Max) and restricted (middle 52%) dimensions (rMin, rMax).

	Min	Max	rMin	rMax	Multiplier
Hue	.403	.555	.443	.515	.025
Size	15	50	24.2	40.8	6

Note. All training stimuli values were set between the restricted minimum and maximum values while all test stimuli were allowed to vary between the extended minimum and maximum values. The base prototype values varied along the whole dimension in the High Variability group, but was restricted to the middle 52% of values in the Low Variability group. The category prototype and all test stimuli were created by multiplying the multiplier by the distortion level and adding or subtracting to the base prototype value.

For each participant, a ‘base’ prototype was first created which contained randomly chosen hue and size values for each of the 9 circles (in Figure 2, the base prototype would be at the intersection of the two dimensions). The color and size values for the base prototype could be any value within the respective ranges. The category prototypes (P1 and P2 in Figure 2) were then constructed from the base prototype. For each participant, one category (left/right) was randomly chosen to have larger and the other smaller circles, and one category was chosen to have bluer and the other greener circles. From the base prototype, the color (hue) and size values of all circles were distorted in opposing directions to the same degree, such that one category’s size values were all larger than the other category’s, and one category’s hue values were all larger than the other category’s (see Figure 2). The exact degree of distortion was determined by multiplying the distortion level (arbitrary level of 0.8) by the feature multiplier (see Table 1) and then adding or subtracting these values from the base prototype values to form the category prototypes.

These category prototypes then formed the basis for creating the 120 training stimuli, which all contained the same color and size values but different (randomized) location coordinates within its cell in the stimulus grid. Thus, all the category exemplars were unique but similar to each other in terms of the relevant dimensions. To make the training phase even more difficult so that participants would not immediately work out a rule on both dimensions, each training stimulus had 2/9 of its color values and 2/9 of its size values (randomly and independently selected for each stimulus) swapped with values in the other category prototype. This effectively meant that the category exemplars seen during training were more similar to each other than the Train stimuli seen on test. Randomizing the locations of the circles and swapping the color and size values served to discourage participants from focusing on a single circle in discrimination, and also added some noise to make the initial discrimination harder. For all groups, the color and size values for the category prototypes and training stimuli were restricted to the middle 52% of values (see Table 1).

This was done because we wanted to ensure that the test stimuli were more extreme than the training stimuli to allow an adequate assessment of generalization along each dimension.

The test stimuli (Train, Near1, Near2, Near3 and Far, see Figure 2 and Table 2) were spaced at regular intervals (arbitrary distortion level 0.6) and were created by distorting the category prototype. The test stimuli were created in the same way as the training stimuli (varying hue and size of each of the 9 circles), except there was no swapping of values for the other prototype and only one dimension was varied at a time. This meant that there were 20 different test stimuli (5 per dimension varied, for each of the two categories). Four sets of the 20 test stimuli were created, each with randomized location values amounting to 80 test stimuli in total. For the Consistent group, when one dimension was varied, the other dimension was set at the values of the category prototype. For the Inconsistent group, the non-varied dimension was set at the values of the base prototype. Since the base prototype was the starting point to create the two category prototypes, the base prototype values were effectively at the midpoint of both categories, and therefore its values were non-diagnostic of category membership.

In the Inconsistent group, participants either saw non-diagnostic information on the non-varied dimension that was highly variable (color: some very blue and some very green circles, or size: some very large and some very small circles) or less variable (color: all circles mostly bluey-green, or size: all circles a medium size). For Experiment 1 and 2, all participants had High Variability in their base prototype, to provide the best chance of our test manipulation disrupting rule use.

Table B. Average distortion values from the base prototype for each test stimulus.

Group	Dimension	LEFT CATEGORY					RIGHT CATEGORY				
		Far	Near3	Near2	Near1	Train	Train	Near1	Near2	Near3	Far
Pilot/Exp 1: Group Consistent	Col	-3.2	-2.6	-2	-1.4	-.8	+.8	+1.4	+2	+2.6	+3.2
	Size*	-.8	-.8	-.8	-.8	-.8	+.8	+.8	+.8	+.8	+.8
	Size	-3.2	-2.6	-2	-1.4	-.8	+.8	+1.4	+2	+2.6	+3.2
	Col*	-.8	-.8	-.8	-.8	-.8	+.8	+.8	+.8	+.8	+.8
Pilot/Exp 1: Group Inconsistent	Col	-3.2	-2.6	-2	-1.4	-.8	+.8	+1.4	+2	+2.6	+3.2
	Size*	0	0	0	0	0	0	0	0	0	0
	Size	-3.2	-2.6	-2	-1.4	-.8	+.8	+1.4	+2	+2.6	+3.2
	Col*	0	0	0	0	0	0	0	0	0	0
Exp 2: Group Consistent	Att	-3.2	-2.6	-2	-1.4	-.8	+.8	+1.4	+2	+2.6	+3.2
	Unatt*	0	0	0	0	0	0	0	0	0	0
	Unatt	-3.2	-2.6	-2	-1.4	-.8	+.8	+1.4	+2	+2.6	+3.2
	Att*	-.8	-.8	-.8	-.8	-.8	+.8	+.8	+.8	+.8	+.8
Exp 2: Group Inconsistent	Att	-3.2	-2.6	-2	-1.4	-.8	+.8	+1.4	+2	+2.6	+3.2
	Unatt*	0	0	0	0	0	0	0	0	0	0
	Unatt	-3.2	-2.6	-2	-1.4	-.8	+.8	+1.4	+2	+2.6	+3.2
	Att*	0	0	0	0	0	0	0	0	0	0

Note. The key difference between test groups was whether the non-varied dimension (*) contained information about the correct category (Consistent) or not (Inconsistent). Numbers indicate the degree of distortion from the base prototype (distortion level 0), which is the stimulus in the middle of the categories, with negative numbers indicating that the stimulus belongs to the left category and positive numbers indicating that the stimulus belongs to the right category. In Experiments 1 and 2 there was an attended and unattended dimension which could either be color or size of the circles. The key difference between Experiments 1 and 2 was that the critical test stimuli varying the attended dimension was equated between Consistent and Inconsistent groups in Experiment 2 but not in Experiment 1.

Appendix B: Additional Pilot Experiment

An additional pilot experiment was conducted with no instructional manipulation. This experiment was exploratory since it was unknown whether participants would easily form relational rules on both dimensions, on one of the dimensions, or neither dimension. A detailed questionnaire was included at the end of the experiment to assess which dimensions participants found useful and whether they could readily identify differences between the categories in terms of color and size.

Method

Participants

One hundred and thirty-three University of Sydney students (*M* age = 20.6, *SD* = 4.52, 105 females) participated in this experiment in exchange for partial course credit or payment (AUD$15/hour). Participants were randomly allocated to the Consistent (n = 68) or Inconsistent group (n = 65). Participants who indicated that they were colorblind were excluded from the analyses (6 participants).

Procedure

The stimuli, apparatus, and procedure were identical to the experiments reported in the manuscript except there was no attention manipulation, no manipulation check, and the order of the 3AFC and 2AFC questions were asked in randomized order (color and then size, or size and then color).

Results

Exclusion Criteria

To ensure that the data analysis included only participants who learned something about the categories, similar to Livesey and McLaren (2009), participants who scored less than or equal to 55% in the last half of training were excluded (36 participants, 27.1% of the sample). After

applying this criterion, a total of 91 participants remained (49 in the Consistent and 42 in the Inconsistent group).

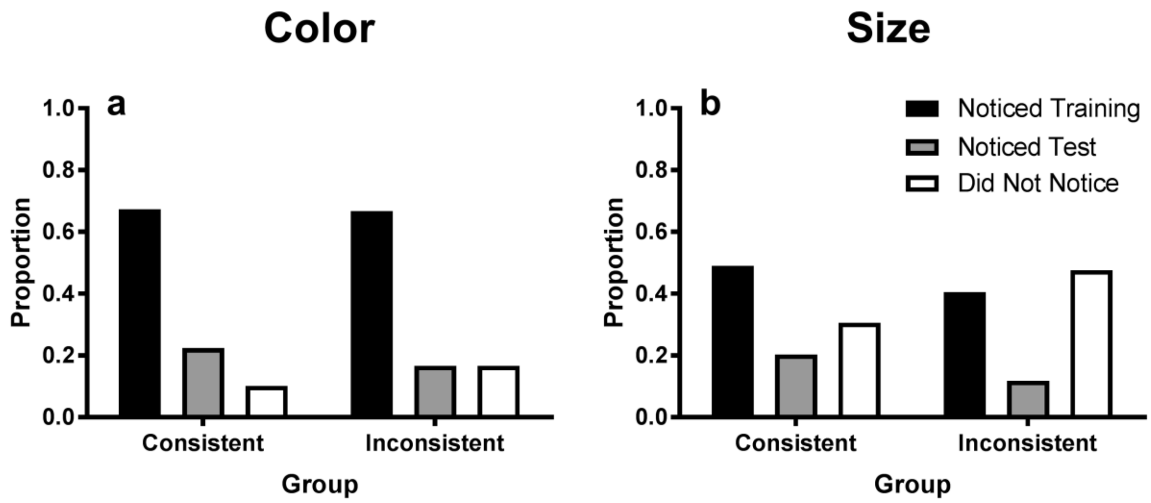

Fig A. Self-report answers from the questionnaire in the pilot experiment.
Proportion of participants who selected each option reporting when they noticed a difference between the categories for each dimension in the pilot experiment.

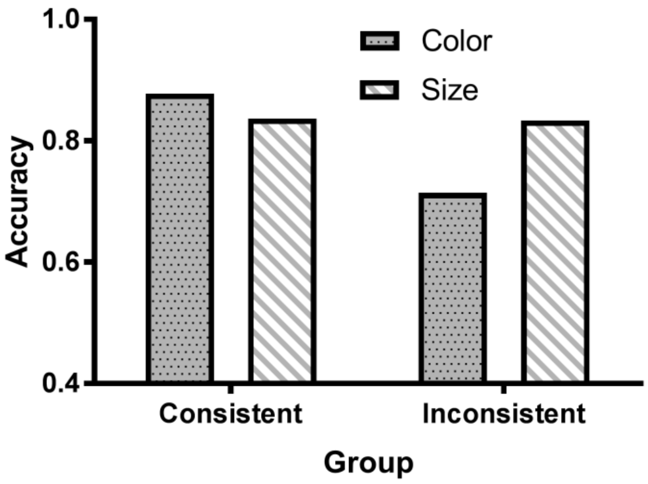

Fig B. Accuracy for the 2AFC rule-identification question in the pilot experiment.

Table C. Number of participants who reported noticing a difference in each dimension during training vs. during test/not at all in the pilot experiment.

		Size		TOTAL
		Noticed in Training	Did not notice in training	
Color	Noticed in Training	19	42	61
	Did not notice in training	22	8	30
TOTAL		41	50	91

probability (noticing size) = .451 (41/91)
probability (noticing size | noticing color) = .311 (19/61)
probability (noticing color) = .670 (61/91)
probability (noticing color | noticing size) = .463 (19/41)

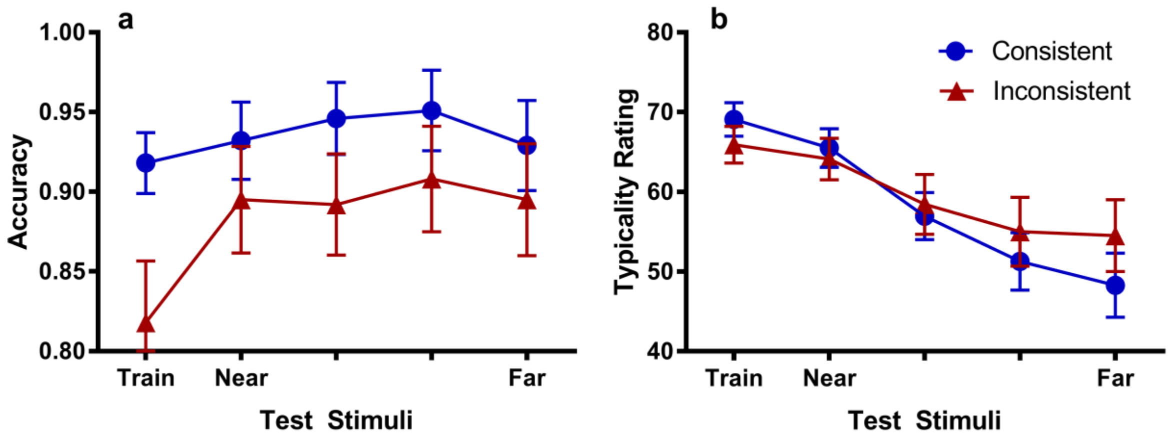

Fig C. Categorization accuracy (A) and typicality ratings (B) for test stimuli varying the "more attended" dimension.
Error bars represent the standard error of the mean.

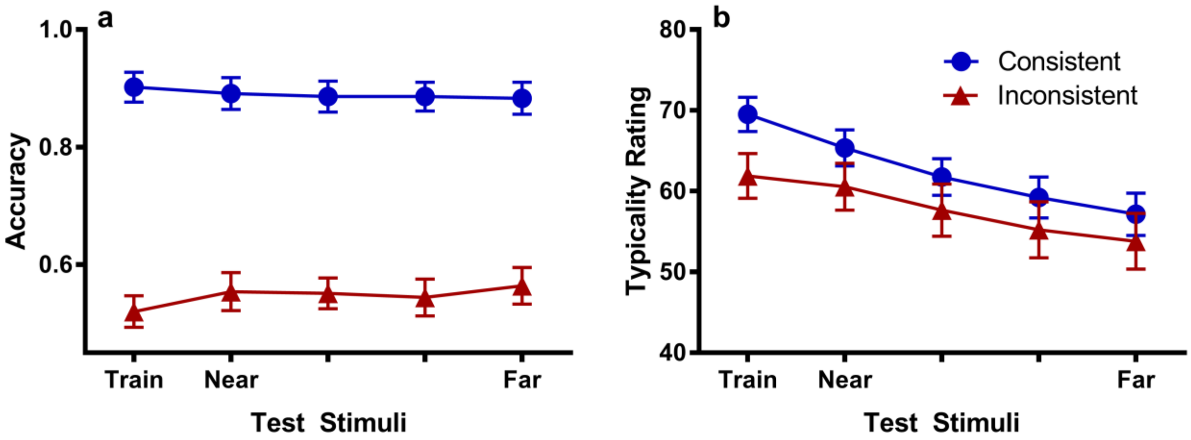

Fig D. Categorization accuracy (A) and typicality ratings (B) for test stimuli varying the "less attended" dimension.

Error bars represent the standard error of the mean.

Appendix C: Scatterplots of Training and Test Accuracy

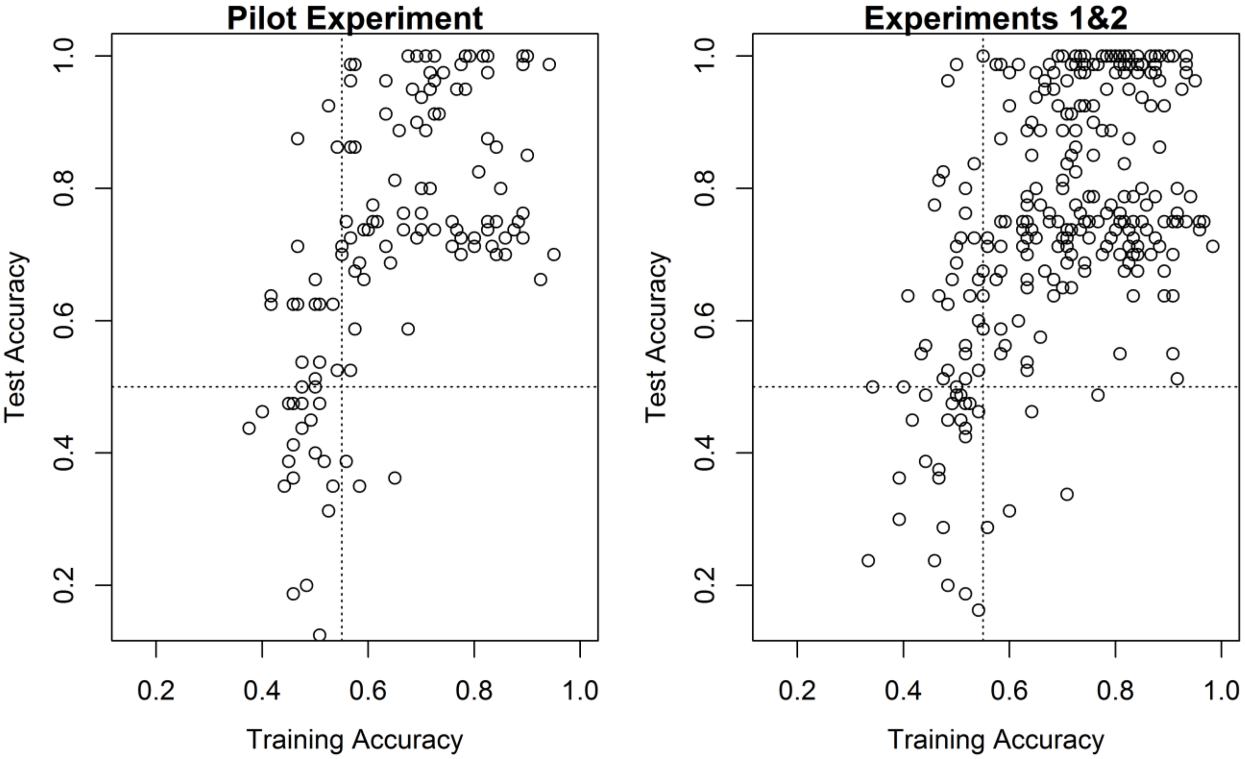

Fig E. Scatterplot of training and test accuracy for the pilot experiment and Experiments 1 and 2 combined.
The 55% training criterion is marked with a vertical dotted line, and 50% test accuracy is marked with a horizontal dotted line.

Appendix D: Test Results for the Unfolded Dimension

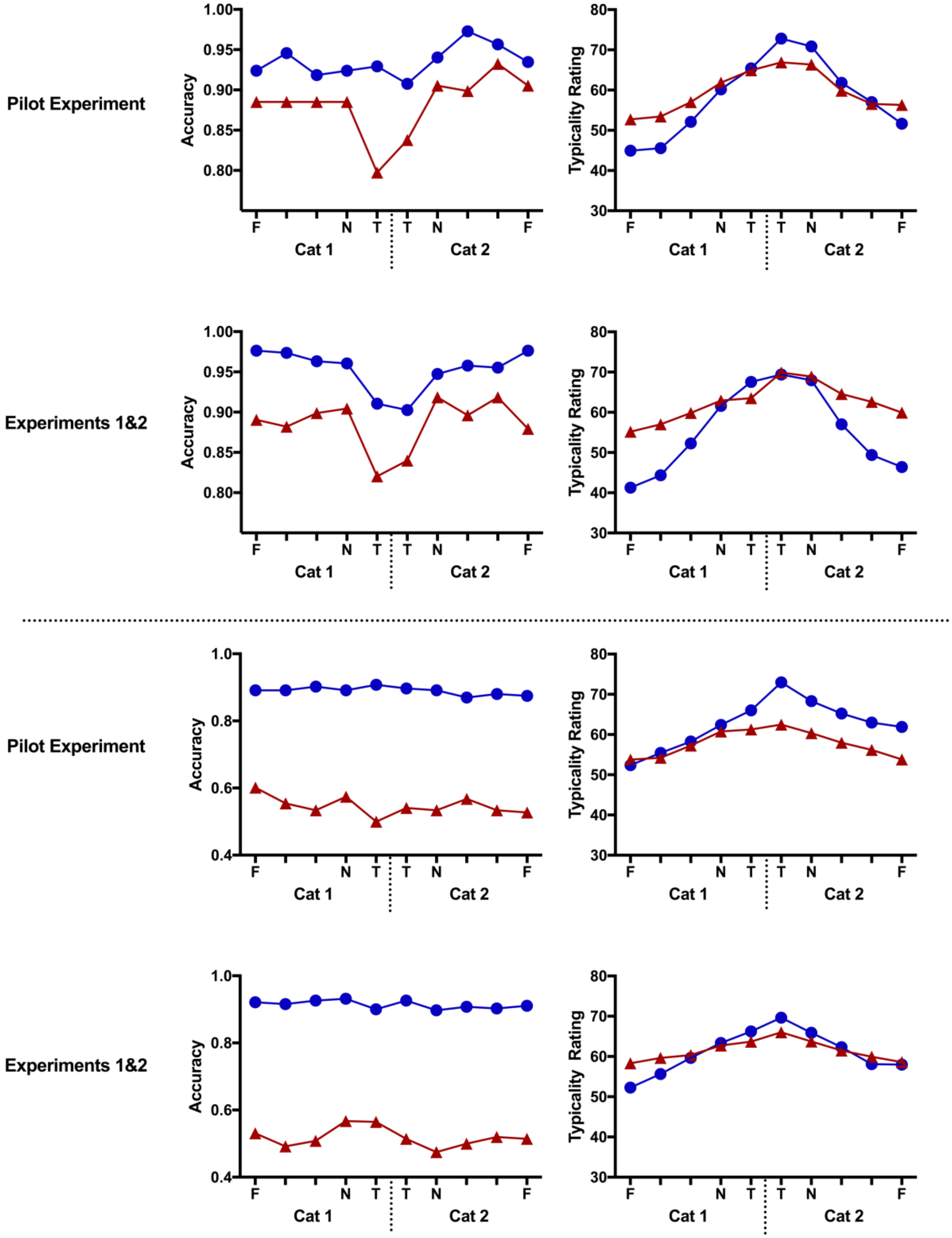

Fig F. Test results for the unfolded dimension.

Categorization accuracy (left panels) and typicality ratings (right panels) for the unfolded attended (upper panels) and unattended (lower panels) dimensions in the pilot experiment, and Experiments 1 and 2 combined (reported in the manuscript).
